# Polydatin Attenuates Cisplatin-Induced Acute Kidney Injury via SIRT6-Mediated Autophagy Activation

**DOI:** 10.1155/2022/9035547

**Published:** 2022-09-16

**Authors:** Zhen Li, Lu Zhou, Yiwei Du, Huirong Li, Lan Feng, Xiangnan Li, Xiao Han, Hongbao Liu

**Affiliations:** Department of Nephrology, Tangdu Hospital, Air Force Military Medical University (Fourth Military Medical University), Xi'an 710038, China

## Abstract

In the treatment of malignant tumors, the effectiveness of cisplatin (CP) is limited by its nephrotoxicity, leading to cisplatin-induced acute kidney injury (CP-AKI). Polydatin (PD) has been demonstrated to regulate autophagy in tumors, sepsis, and diabetes. We have recently confirmed that PD attenuated CP-AKI by inhibiting ferroptosis, but it is not clear whether PD can regulate autophagy to protect from CP-AKI. The purpose of this study was to investigate the effect of PD on autophagy in CP-treated HK-2 cells and CP-AKI mouse models, exploring the role of sirtuin 6 (SIRT6) upregulated by PD. In this study, the blocking of autophagy flux was observed in both CP-treated HK-2 cells in vitro and CP-AKI mouse models in vivo, whereas this blocking was reversed by PD, which was characterized by the increase of autophagy microtubule-associated protein light chain 3 II expression and autophagolysosome/autophagosome ratio and the decrease of p62 expression. Furthermore, PD also significantly increased the expression of SIRT6 in vivo and in vitro. The protective effect of PD manifested by the stimulating of autophagy flux, with the reducing of inflammatory response and oxidative stress, which included downregulation of tumor necrosis factor-*α* and interleukin-1*β*, decreased activity of myeloperoxidase and content of malondialdehyde, and increased activity of superoxide dismutase and level of glutathione, both in vivo and in vitro, was reversed by either inhibition of autophagy flux by chloroquine or downregulation of SIRT6 by OSS-128167. Taken together, the present findings provide the first evidence demonstrating that PD exhibited nephroprotective effects on CP-AKI by restoring SIRT6-mediated autophagy flux mechanisms.

## 1. Introduction

As the mainstay in the treatment of various types of tumors, the application of cisplatin (CP) is hindered by dose limitation due to its nephrotoxicity, such as acute kidney injury (AKI) in 20%-30% of patients [1]. However, so far, there is still a lack of effective drugs to treat Cis-AKI clinically. The pathogenesis of CP-induced AKI (CP-AKI) is complex and multifactorial, including autophagy, which has been proved to exert a nephroprotective role in experimental models of CP-AKI by using both pharmacological and genetic approaches in recent years [2]. Autophagy has always been regarded as a double-edged sword in tumor therapy [1, 3], so when targeting autophagy as a nephroprotective strategy in CP-AKI, its effect on tumors should be considered.

Polydatin (PD, C_20_H_22_O_8_), a glucoside of resveratrol, known as a natural active ingredient extracted from *Polygonum cuspidatum* Sieb. et Zucc., is used for both medication and food, which plays a multitarget protective role in AKI by antiapoptosis, anti-inflammation, antioxidative stress, antiferroptosis, and regulation of autophagy [4–12]. What is more, PD has also been proved to play an antitumor effect by inhibiting cell viability, migration, and invasion of different tumor cells [13–15]. PD has been clinically used to treat multiple diseases, showing its safety in human application [16–18], which suggests that it can be used as a promising alternative compound for treating CP-AKI in cancer patients.

In recent years, the relationship between PD and autophagy has attracted considerable attention in different diseases, including sepsis [5, 19, 20], ischemia-reperfusion (I/R) injury [21–23], diabetes [24, 25], atherosclerosis [26], Parkinson's disease [27], and tumors [13, 28–30]. PD has also been verified to play a nephroprotective role by regulating autophagy in sepsis-induced AKI [5, 6] and diabetic nephropathy [25]. In our last study, PD can alleviate CP-AKI by inhibiting ferroptosis [4], but whether autophagy is involved in the nephroprotection of PD on CP-AKI has not been reported yet.

Sirtuin 6 (SIRT6) belongs to a nicotinamide adenine dinucleotide (NAD^+^)-dependent deacetylase, which plays a protective role in different organ injuries by regulating autophagy [31]. Overexpression of SIRT6 can alleviate CP-AKI [32], but the relationship between SIRT6 and autophagy in CP-AKI remains a mystery. Recently, PD has been found to protect septic myocardial injury by promoting SIRT6-activated autophagy [19]. Our previous studies confirmed the renal protective effect of PD through antioxidative stress and antiferroptosis in I/R-induced AKI (I/R-AKI) and CP-AKI [4, 9, 11]. Based on the above evidence, we hypothesized that PD may attenuate CP-AKI through SIRT6-mediated autophagy.

Therefore, this study intends to investigate the correlation between autophagy and SIRT6 activation in the nephroprotection of PD in CP-AKI model both in vivo and in vitro, which may provide reliable evidence for the research and development of nephroprotective drugs in the clinical treatment of AKI.

## 2. Materials and Methods

### 2.1. Chemicals and Reagents

Cisplatin (CP, Cat. No. MB1055) and Polydatin (PD, Cat. No. MB5448) were purchased from Meilun Biotech (Dalian, China). Chloroquine (CQ, Cat. No. C6628) was bought from Sigma-Aldrich (St. Louis, MO, USA). Dimethyl sulfoxide (DMSO, Cat. No. D8371) was gained from Beijing Solarbio Science & Technology Co, Ltd. Rapamycin (RAP, AV-22989, Cat. No. S1039) and OSS-128167 (SIRT6-IN-1, Cat. No. S8627) were purchased from Selleck Chemicals (Houston, TX, United States). Dulbecco's modified Eagle's medium (DMEM, Cat. No. SH30243.01) was bought from Cytiva. Fetal bovine serum (FBS, Cat. No. IC-1905) was gained from InCellGenE LLC. Phosphate-buffered saline (PBS, Cat. No. G4202), fluorescein (FITC) TUNEL cell apoptosis detection kit (Cat. No. G1501), and DAPI fluorescence staining kit (Cat. No. G1012) were purchased from the Wuhan Servicebio Technology Co Ltd. Additionally, myeloperoxidase (MPO, Cat. No. A044-1-1), malondialdehyde (MDA, Cat. No. A003-1-2), superoxide dismutase (SOD, Cat. No. A001-3-2), and glutathione (GSH, Cat. No. A006-2-1) test kits were offered by the Nanjing Jiancheng Bioengineering Institute (Nanjing, China).

### 2.2. Cell Culture and Treatment

**Figure 1:**
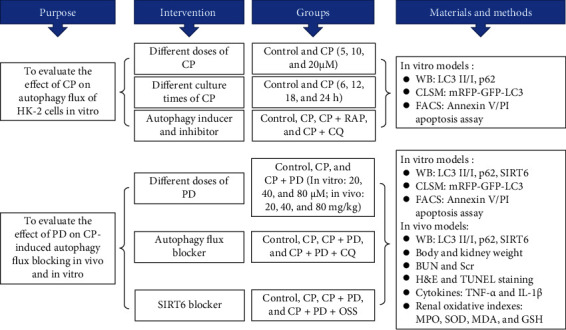
Experimental design for various treatment groups. CP: cisplatin (in vitro: 20 *μ*M; in vivo: 20 mg/kg, i.p.); PD: polydatin (in vitro: 40 *μ*M, 2 h before induction with CP; in vivo: 40 mg/kg, i.p., 1 h before and 24 h after CP); RAP: rapamycin (in vitro: 50 nM, 4 h before induction with CP); CQ: chloroquine (in vitro: 20 *μ*M, 4 h before induction with CP; in vivo: 60 mg/kg, i.p., 1 h before and 24 h after CP); OSS: OSS-128167 (in vitro: 50 *μ*M, 0.5 h before induction with CP; in vivo: 50 mg/kg, i.p., 1 h before CP); SIRT6: sirtuin 6; WB: western blot; LC3: autophagy microtubule-associated protein light chain 3; p62: sequestosome 1 (SQSTM1); CLSM: confocal laser scanning microscope; FACS: fluorescence-activated cell sorting; BUN: blood urea nitrogen; Scr: serum creatinine; H&E: hematoxylin-eosin; TUNEL: terminal deoxynucleotidyl transferase dUTP nick-end labeling; TNF-*α*: tumor necrosis factor-*α*; IL-1*β*: interleukin-1*β*; MPO: myeloperoxidase; MDA: malondialdehyde, SOD: superoxide dismutase; GSH: glutathione.

Human proximal tubular epithelial cells (HK-2 cells), obtained from the China Center for Type Culture Collection (GDC0152, Wuhan, China), were cultured in DMEM medium containing 10% FBS and 100 units of antibiotics (streptomycin and penicillin) per ml at 37°C. The exponentially growing HK-2 cells were inoculated in six-well culture plate with 2 to 4 × 10^5^ cells/well and cultured for 1 day before each experiment. After treating HK-2 cells with CP in dose gradient (5 *μ*M, 10 *μ*M, and 20 *μ*M) and time gradient (6 h, 12 h, 18 h, and 24 h), according to the protein expression of LC3 II and p62, the cells treated with CP in a dose of 20 *μ*M for 24 h were determined for the follow-up experiment. To investigate the effect of CP on autophagy of HK-2 cells, the cells were pretreated with CQ (20 *μ*M) or RAP (50 nM) for 4 h before induction with CP (20 *μ*M) for 24 h. To evaluate the role of PD on autophagy, HK-2 cells were pretreated with PD in a dose gradient (20 *μ*M, 40 *μ*M, and 80 *μ*M) for 2 h before induction with CP (20 *μ*M) for 24 h. According to the change of the protein expression of LC3 II and p62, and cell apoptosis rate in HK-2 cells, a 40 *μ*M dose of PD was determined as the best dose. Therefore, the following experiments divided the cells into four groups: control group, CP (20 *μ*M) group, CP + PD (40 *μ*M) group, and CP + PD + CQ (20 *μ*M) group. To clarify the role of SRIT6 in PD on the autophagy flux of CP-treated HK-2 cells, OSS-128167 (50 *μ*M), a SIRT6 selective inhibitor, was used instead of CQ to pre-treat the cells for 0.5 h before induction with CP (20 *μ*M) for 24 h. Experimental design in vitro is shown in Figure 1.

### 2.3. Western Blot (WB) Analysis

Equal amounts of protein from both cultured HK-2 cells and renal tissue lysates were loaded and separated using 10% sodium dodecyl sulfate (SDS) polyacrylamide gel and transferred to a polyvinylidene fluoride (PVDF) membrane. The membranes were blocked in 5% nonfat dry milk (diluted in TBST with 0.1% Tween-20) for 1 h at room temperature. The membrane was incubated overnight at 4°C of light chain 3A/B (LC3A/B, 1 : 1000, Cell Signaling Technology, Cat. No.#4108), SIRT6 (1 : 1000, Cell Signaling Technology, Cat. No. #12486), sequestosome 1 (SQSTM1/p62, 1 : 10000, Abcam, Cat. No. ab109012), or GAPDH (1 : 1000, Servicebio Technology, Cat. No. GB12002) antibodies which were appropriately diluted in 1 × TBST containing 5% nonfat dry milk, followed by peroxidase-conjugated secondary antibody. The immune complexes were visualized with appropriate horseradish peroxidase-conjugated secondary antibodies (1 : 2000, Cat. No. SA-10010 and SA-10011, InCellGenE LLC) and enhanced chemiluminescence system (Cat. No. IC-5008-100, InCellGenE LLC).

### 2.4. Transfection of mRFP-GFP-LC3 Adenovirus

Cells were transfected with monomeric red fluorescent protein (mRFP)-green fluorescent protein (GFP)-LC3 adenovirus (Cat. No. HB-AP2100001, Hanheng Biotechnology, Shanghai, China) supplemented with half the volume of medium after 4 h and replaced with fresh medium after 6 h to continue the culture. Different groups were treated with corresponding drugs, and the cells were photographed under a confocal laser scanning microscope (CLSM) 24 h after adding CP.

### 2.5. Apoptosis Assay

Apoptosis was detected using an Annexin V-fluorescein isothiocyanate (FITC) apoptosis detection kit (Cat. No. 556547, BD Pharmingen, USA) following the manufacturer's instructions. 5 *μ*l Annexin V-FITC and 2 *μ*l propidium iodide (PI) were added into the single-cell suspension, which was incubated in the dark for 15 min, and then the cells were analyzed by fluorescence-activated cell sorting (FACS, Beckman, USA).

### 2.6. Animals and Experimental Protocol

Male C57BL/6 mice (8-10 weeks old) were purchased from the Animal Center of Air Force Military Medical University (Fourth Military Medical University). All animal experiments were conducted in strict accordance with the Guidelines of Health and guidelines for use, and were permitted by the Animal Welfare and Ethics Institution of the Tangdu Hospital, Air Force Military Medical University (IACUC approval number 202003-100). According to previous reports from us and other authors [2, 4], a single intraperitoneal injection of CP at a dose of 20 mg/kg is sufficient to induce AKI in mice. To select the best dose of PD in CP-AKI mice, the animals were randomly divided into five experimental groups (*n* = 6-8 each) as follows: control (equivalent saline containing 1% DMSO, i.p.) group, CP (20 mg/kg dissolved in saline, i.p.) group, CP + PD low-dose (PD-L, 20 mg/kg dissolved in 1% DMSO, i.p.) group, CP + PD middle-dose (PD-M, 40 mg/kg dissolved in 1% DMSO, i.p.) group, and CP + PD high-dose (PD-H, 80 mg/kg dissolved in 1% DMSO, i.p.) group [11]. To estimate the effect of autophagy flux blocking on renal protection of PD in CP-AKI mice, the animals were divided into the following four groups (*n* = 6-8 each): control group, CP (20 mg/kg, i.p.) group, CP + PD (40 mg/kg, i.p.) group, and CP + PD + CQ (60 mg/kg, i.p.) group [33]. To evaluate the role of SRIT6 in PD on the autophagy flux in CP-AKI mice, OSS-128167 (50 mg/kg, i.p.) [34] was used to replace CQ in the previous groups (*n* = 6-8 each). Mice were injected with CP once; PD or CQ was given 1 h before and 24 h after CP. OSS-128167 was administered only 1 h before CP. Experimental design in vivo is shown in Figure 1. The whole blood and kidneys were collected when animals were ethically killed by dislocating their spines at 48 h after CP injection.

### 2.7. Measurement of Body Weight and Kidney Index

The body weight of each mouse in different groups was recorded at 1 h before and 48 h after CP injection. The kidney tissue was taken and weighed, and the kidney index was calculated according to the formula: kidney weight (g)/body weight (g) × 100%.

### 2.8. Blood Physiochemical Assays

Blood samples were allowed to stand at room temperature for 4 h. After waiting for coagulation, they were centrifuged at 4000 rpm for 10 min to acquire the serum sample. Mouse serum creatinine (Scr) and blood urea nitrogen (BUN) were detected using the creatinine determination kit (C011-2-1, Nanjing Jiancheng, China) and urea determination kit (C013-2-1, Nanjing Jiancheng, China), respectively.

### 2.9. Preparation of Kidney Tissue Homogenate

The kidney tissues were accurately weighed and homogenized on an ice bath with a tissue homogenizer according to the ratio of kidney weight (g) and 50 mmol/l phosphate buffer (pH 7.4) volume (ml) = 1 : 9. Centrifuge at 1500 g for 20 min and collect the supernatant as the sample to be tested immediately.

### 2.10. Enzyme-Linked Immunosorbent Assay (ELISA)

The levels of tumor necrosis factor-*α* (TNF-*α*) and interleukin-1*β* (IL-1*β*) in renal tissue homogenate were evaluated with a commercially available ELISA kit (R&D Systems, USA), according to the manufacturer's recommendation. All standards and samples were measured in duplicate.

### 2.11. Measurement of Renal Oxidative Indexes

The kidney supernatant was used to measure the activity of MPO and SOD, and the content of MDA and GSH followed the commercial kit instructions by using a spectrophotometer (Spectrophotometer DU640, Beckman Coulter, Fullerton, CA). All levels were expressed as U/mg protein, nmol/mg protein, or mg/g protein, respectively.

### 2.12. Renal Histopathology and Terminal Deoxynucleotidyl Transferase dUTP Nick-End Labeling (TUNEL) Assay

Fresh kidney tissue was rinsed with frozen stroke physiological saline, fixed in 10% neutral buffered formalin overnight, embedded in paraffin, and cut into 4*-μ*m-thick sections for hematoxylin-eosin (H&E) staining and TUNEL fluorescent staining according to the manufacturer's instructions. Renal tubular epithelial cells with the following histopathological changes were considered injured: loss of brush border, tubular dilation and disruption, cast formation, and cell lysis. The kidney tissue damage was performed by renal pathologists in a blinded fashion and scored by calculating the percentage of damaged tubules: 0 none; 1, <25%; 2, 25-50%; 3, 50-75%; and 4, >75%. The scores of at least 10 randomly selected areas per mouse kidney were averaged and used as the scores of the individual mouse kidneys.

### 2.13. Statistical Analysis

All data are presented as mean ± standard deviation (SD) of at least three independent experiments. Statistical analysis of data was performed with SPSS 19, and *P* values were determined using Student's *t-test* and one-way analysis of variance (ANOVA) for two independent samples. Immunofluorescence and grayscale analysis of western blot bands were performed semiquantitative analysis using ImageJ. *P* < 0.05 was statistically significant.

## 3. Results

### 3.1. Autophagy Flux Was Restrained in Cisplatin-Treated HK-2 Cells

**Figure 2:**
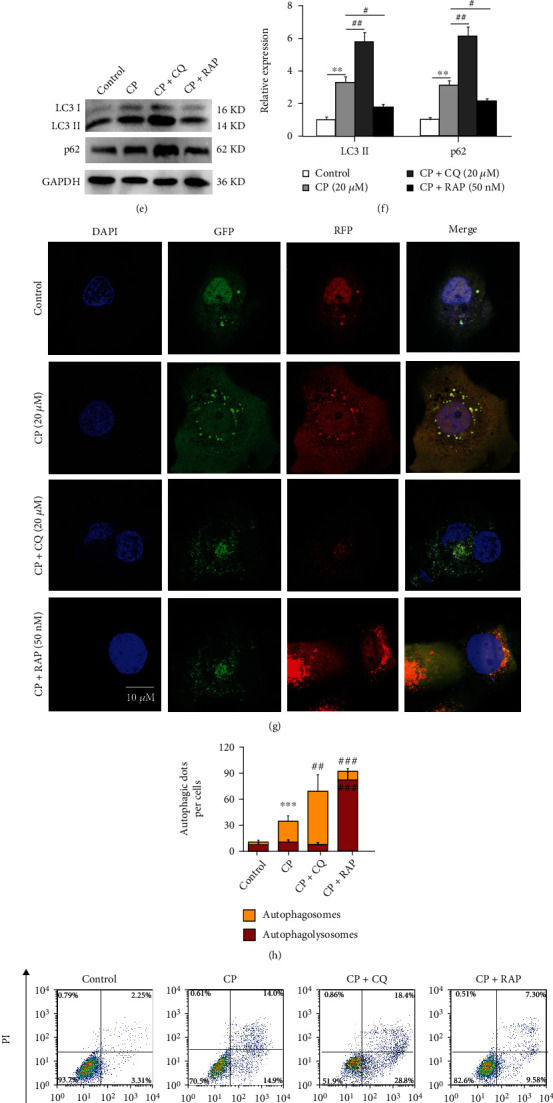
Cisplatin-induced autophagy flux blocking in cultured HK-2 cells. (a and b) Western blot of LC3 and p62 in HK-2 cells treated with different doses of CP (5 *μ*M, 10 *μ*M, and 20 *μ*M) for 24 h, and the semiquantitative analysis of LC3 II/GAPDH and p62/GAPDH were shown. (c and d) Western blot of LC3 and p62 in HK-2 cells treated with 20 *μ*M CP at different time (6 h, 12 h, 18 h, and 24 h), and the semiquantitative analysis of LC3 II/GAPDH and p62/GAPDH were shown. (e and f) Western blot of LC3 and p62 in CP (20 *μ*M)-treated HK-2 cells supplemented with CQ (20 *μ*M) or RAP (50 nM), and the semiquantitative analysis of LC3 II/GAPDH and p62/GAPDH were shown. (g and h) CLSM image of HK-2 cells expressing mRFP-GFP-LC3. HK-2 cells transfected with adenovirus harboring tandem fluorescent mRFP-GFP-LC3 for 24 h were subjected to CP (20 *μ*M) combined with CQ (20 *μ*M) or RAP (50 nM). The nuclei were labeled with DAPI (blue), the GFP dots were green, and the mRFP dots were red. Scale bar = 10 *μ*m. The semiquantitative analysis of autophagosome (yellow dots in merged images) and autophagolysosome (red only dots in merged images) were shown. (i and j) In vitro survival analysis of HK-2 cells treated CP (20 *μ*M) supplemented with CQ (20 *μ*M) or RAP (50 nM), and bar graph described from the FACS-based Annexin V/PI apoptosis assay. ^∗^*P* < 0.05, ^∗∗^*P* < 0.01, and ^∗∗∗^*P* < 0.001 vs. control; ^#^*P* < 0.05, ^##^*P* < 0.01, and ^###^*P* < 0.001 vs. CP (*n* = 6). CP: cisplatin; CQ: chloroquine; RAP: rapamycin; LC3: autophagy microtubule-associated protein light chain 3; p62: sequestosome 1 (SQSTM1); PI: propidium iodide; CLSM: confocal laser scanning microscope; FACS: fluorescence-activated cell sorting.

Autophagy flux detection is considered as the “gold standard” to evaluate autophagy level [35], so we analyzed the effect of CP on the expression of autophagy-related genes LC3 and p62 in HK-2 cells. The results of western blot showed that CP led to the upregulation of the LC3 II and p62 protein expression in HK-2 cells in a dose-dependent manner, and this change was most significant at the dose of 20 *μ*M CP (Figures 2(a) and 2(b)). Therefore, we chose 20 *μ*M CP to treat the cells and observed the effects of different culture times (6, 12, 18, and 24 h) on the autophagy flux of HK-2 cells. Similarly, LC3 II and p62 expression were also increased in a time-dependent manner by CP, and this change was most significant at 24 h of culture, about 3.48- and 4.17-fold as compared to the control group (Figures 2(c) and 2(d)). These findings indicated that the autophagy degradation of HK-2 cells was interfered by CP, and the subsequent experiments in this study were carried out when the HK-2 cells were treated with CP at a dose of 20 *μ*M for 24 h. Then, we introduced CQ (an autophagy inhibitor) or RAP (an autophagy activator) into CP-treated HK-2 cells and evaluated the changes of autophagy flux by the above protein expression assay and mRFP-GFP-LC3 transfection method. WB results showed that the LC3 II and p62 expression increased by CP were promoted by CQ and decreased by RAP (Figures 2(e) and 2(f)). mRFP-GFP-LC3 is a widely used autophagic indicator with a yellow LC3 signal when in the autophagosome (with both GFP and RFP signals) and a red LC3 signal when in the autophagolysosome due to the acidic milieu that quenches GFP signal. As shown in Figures 2(g) and 2(h), compared with the control, CP induced the increase of yellow puncta (named YFP), suggesting the blockage of autophagy flux from the autophagosome to the autophagolysosome. Furthermore, the CP-increased YFP/RFP ratio was further aggravated by CQ and reversed by RAP (Figures 2(g) and 2(h)). Concomitantly, the FACS analysis of Annexin V and PI staining showed that the increased apoptosis induced by CP was worsened by CQ and alleviated by RAP (Figures 2(i) and 2(j)). These findings suggested that CP-induced autophagy flux blocking had adverse effects on HK-2 cells.

### 3.2. PD Attenuated Cisplatin-Induced Apoptosis of HK-2 Cells by Restoring the Autophagy Flux

**Figure 3:**
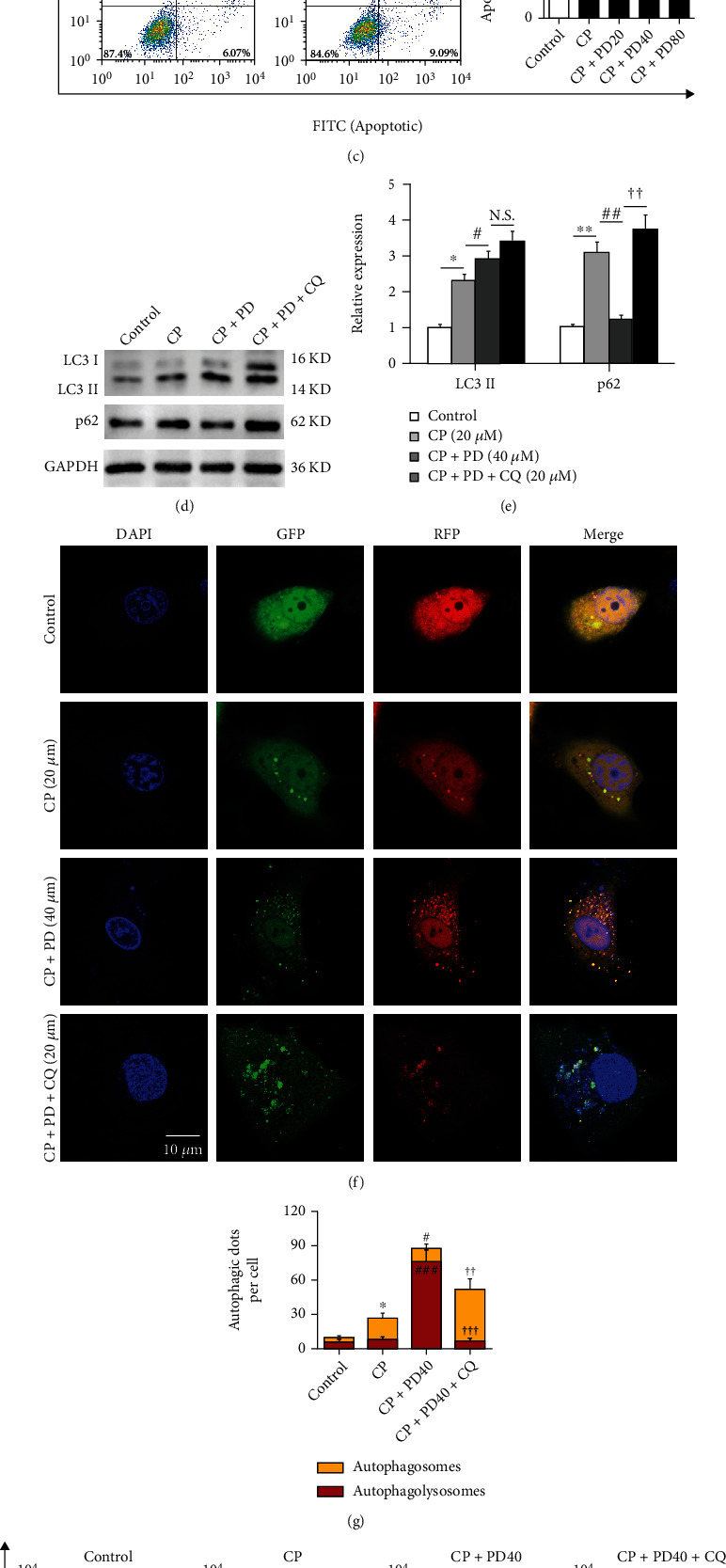
PD attenuated cisplatin-induced apoptosis of HK-2 cells by restoring the autophagy flux. (a and b) Western blot of LC3 and p62 in CP (20 *μ*M)-treated HK-2 cells supplemented with different doses of PD (20 *μ*M, 40 *μ*M, and 80 *μ*M), and the semiquantitative analysis of LC3 II/GAPDH and p62/GAPDH were shown. (c) In vitro survival analysis of HK-2 cells treated CP (20 *μ*M) supplemented with different doses of PD (20 *μ*M, 40 *μ*M, and 80 *μ*M), and bar graph described from the FACS-based Annexin V/PI apoptosis assay. (d and e) Western blot of LC3 and p62 in CP (20 *μ*M)-treated HK-2 cells supplemented with PD (40 *μ*M) or PD plus CQ (20 *μ*M), and the semiquantitative analysis of LC3 II/GAPDH and p62/GAPDH were shown. (f and g) CLSM image of HK-2 cells expressing mRFP-GFP-LC3. HK-2 cells transfected with adenovirus harboring tandem fluorescent mRFP-GFP-LC3 for 24 h were subjected to CP (20 *μ*M) combined with PD (40 *μ*M) and CQ (20 *μ*M). The nuclei were labeled with DAPI (blue), the GFP dots were green, and the mRFP dots were red. Scale bar = 10 *μ*m. The semiquantitative analysis of autophagosome (yellow dots in merged images) and autophagolysosome (red only dots in merged images) were shown. (h and i) In vitro survival analysis of CP (20 *μ*M)-treated HK-2 cells supplemented with PD (40 *μ*M) or PD plus CQ (20 *μ*M), and bar graph described from the FACS-based Annexin V/PI apoptosis assay. ^∗^*P* < 0.05, ^∗∗^*P* < 0.01, and ^∗∗∗^*P* < 0.001 vs. control; ^#^*P* < 0.05, ^##^*P* < 0.01, and ^###^*P* < 0.001 vs. CP; ^†^*P* < 0.05, ^††^*P* < 0.01, and ^†††^*P* < 0.001 vs. CP + PD40 (40 *μ*M) (*n* = 6). CP: cisplatin; PD: polydatin; CQ: chloroquine; LC3: autophagy microtubule-associated protein light chain 3; p62: sequestosome 1 (SQSTM1); PI: propidium iodide; CLSM: confocal laser scanning microscope; FACS: fluorescence-activated cell sorting.

Although it has been proved that PD plays a renal protective role through activating autophagy in sepsis-induced AKI and diabetic nephropathy, the relationship between PD and autophagy in CP-AKI remains unclear. First, we evaluated the effects of different doses of PD (20 *μ*M, 40 *μ*M, and 80 *μ*M) on the autophagy flux in CP-treated HK-2 cells. WB results showed that, compared with the control, CP (20 *μ*M) significantly induced the increase of LC3 II and p62 expression (Figures 3(a) and 3(b)). PD increased the lipid conjugation of free LC3 I to the autophagic membrane-associated LC3 II and decreased the expression of p62 in a dose-dependent manner, with 40 *μ*M and 80 *μ*M being the most significant (Figures 3(a) and 3(b)). Concomitantly, 40 *μ*M and 80 *μ*M doses of PD significantly reduced CP-induced cell apoptosis compared with 20 *μ*M PD (Figure 3(c)), so we chose 40 *μ*M dose of PD for the follow-up experiment. To determine whether PD regulates autophagosome formation or autophagy flux, CQ, known as a lysosomal inhibitor, has been used in the experiments. As expected, in CP-treated HK-2 cells, compared with PD alone, PD combined with CQ (20 *μ*M) increased the expression of LC3 II and p62 (Figures 3(d) and 3(e)). Similarly, the mRFP-GFP-LC3 transfection method showed that compared with CP alone, PD significantly induced the increase of red puncta, but PD combined with CQ significantly increased the yellow puncta (with both GFP and RFP signals), suggesting that PD enhanced not only autophagosome formation but autophagy flux (Figures 3(f) and 3(g)). Concomitantly, the antiapoptosis effect of PD in CP-treated HK-2 cells was significantly reversed by CQ (Figures 3(h) and 3(i)). These results suggested that PD can reverse CP-induced HK-2 cell injury by restoring autophagy flux.

### 3.3. The Inhibition of SIRT6 Reversed the Recovery of PD on Cisplatin-Induced Autophagy Flux Blocking in HK-2 Cells

**Figure 4:**
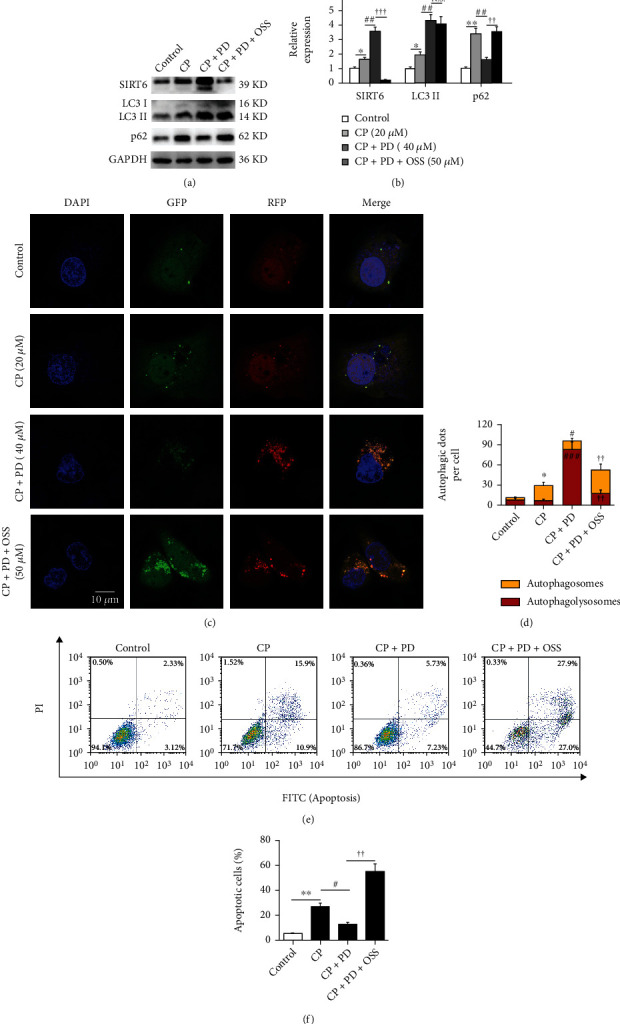
The inhibition of SIRT6 reversed the recovery of PD on cisplatin-induced autophagy flux blocking in HK-2 cells. (a and b) Western blot of SIRT6, LC3, and p62 in CP (20 *μ*M)-treated HK-2 cells supplemented with PD (40 *μ*M) or PD plus OSS-128167 (50 *μ*M), and the semiquantitative analysis of SIRT6/GAPDH, LC3 II/GAPDH, and p62/GAPDH were shown. (c and d) CLSM image of HK-2 cells expressing mRFP-GFP-LC3. HK-2 cells transfected with adenovirus harboring tandem fluorescent mRFP-GFP-LC3 for 24 h were subjected to CP (20 *μ*M) combined with PD (40 *μ*M) and OSS-128167 (50 *μ*M). The nuclei were labeled with DAPI (blue), the GFP dots were green, and the mRFP dots were red. Scale bar = 10 *μ*m. The semiquantitative analysis of autophagosome (yellow dots in merged images) and autophagolysosome (red only dots in merged images) were shown. (e and f) In vitro survival analysis of CP (20 *μ*M)-treated HK-2 cells supplemented with PD (40 *μ*M) or PD plus OSS-128167 (50 *μ*M), and bar graph described from the FACS-based Annexin V/PI apoptosis assay. ^∗^*P* < 0.05, ^∗∗^*P* < 0.01, and ^∗∗∗^*P* < 0.001 vs. control; ^#^*P* < 0.05, ^##^*P* < 0.01, and ^###^*P* < 0.001 vs. CP; ^†^*P* < 0.05, ^††^*P* < 0.01, and ^†††^*P* < 0.001 vs. CP + PD (*n* = 6). CP: cisplatin; PD: polydatin; OSS: OSS-128167; SIRT6: sirtuin 6; LC3: autophagy microtubule-associated protein light chain 3; p62: sequestosome 1 (SQSTM1); PI: propidium iodide; CLSM: confocal laser scanning microscope; FACS: fluorescence-activated cell sorting.

Increasing evidence shows that SIRT6-mediated autophagy has a positive effect on cell survival [31]. To confirm whether SIRT6 is involved in the cytoprotective effect of PD against CP, the CP-induced HK-2 cells were treated with PD combined with or without OSS-128167, an inhibitor of SIRT6, to evaluate the relationship between SIRT6 and autophagy flux. As shown in (Figures 4(a) and 4(b)), CP increased the expression of SIRT6 in cells compared with the control, but PD further significantly increased the SIRT6 expression in CP-treated HK-2 cells, and this effect was dramatically inhibited by OSS-128167. Interestingly, OSS-128167 also inhibited the role of PD in restoring autophagy flux in CP-treated HK-2 cells, according to the increased expression of LC3 II and p62 in WB results (Figures 4(a) and 4(b)). Similarly, the mRFP-GFP-LC3 transfection method showed that, compared with PD alone, PD plus OSS-128167 again caused the blocking of autophagy flux in CP-treated HK-2 cells, according to the decrease of red bolts and the increase of yellow bolts (Figures 4(c) and 4(d)). Furthermore, the apoptosis of CP-treated cells improved by PD was also reversed by OSS-128167 (Figures 4(e) and 4(f)). These data suggested that the inhibition of SIRT6 reversed the recovery of PD on CP-induced autophagy flux blocking in HK-2 cells.

### 3.4. PD Can Improve the Renal Function and Autophagy Flux Blocking of CP-AKI Animals

**Figure 5:**
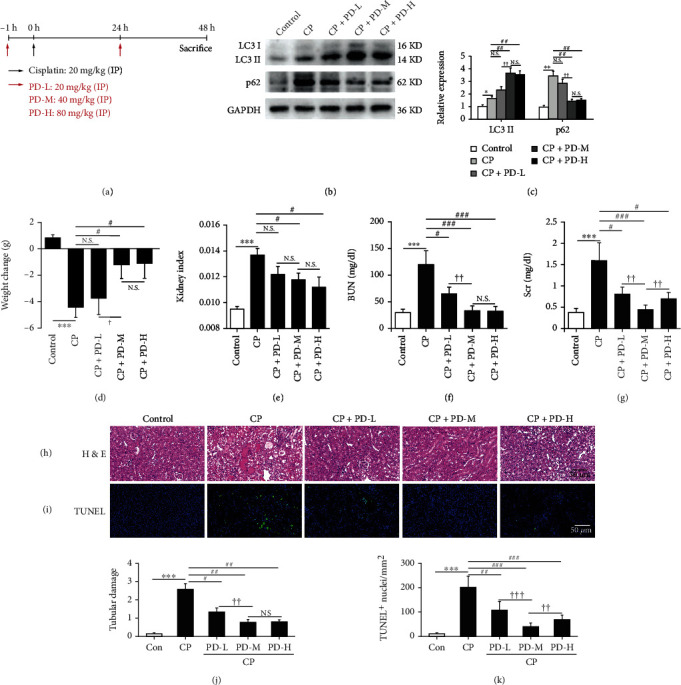
PD can improve the renal function and autophagy flux blocking of CP-AKI mice. (a) Mice pretreated with different doses of PD (20, 40, and 80 mg/kg) were administered with intraperitoneal injections of CP (20 mg/kg), and PD was intraperitoneally reinjected at 24 h after CP injection. They were executed 48 h after the CP injection. (b and c) Western blot of LC3 and p62 in kidneys of CP-AKI mice that received PD-L (20 mg/kg), PD-M (40 mg/kg), PD-H (80 mg/kg), or vehicle (saline with 1% DMSO), and the semiquantitative analysis of LC3 II/GAPDH and p62/GAPDH were shown. (d–g) Body weight changes, kidney index, BUN, and Scr were measured at 48 h after CP injection. (h and i) Histopathology analysis of the kidneys in CP-AKI mice was performed by H&E staining, and the tubular damage was graded. Scale bar = 50 *μ*m. (j and k) Representative TUNEL-stained sections of the kidney. Scale bar = 50 *μ*m. ^∗^*P* < 0.05, ^∗∗^*P* < 0.01, and ^∗∗∗^*P* < 0.001 vs. control; ^#^*P* < 0.05, ^##^*P* < 0.01, and ^###^*P* < 0.001 vs. CP; ^†^*P* <0.05, ^††^*P* <0.01,^†††^*P* <0.001 vs. CP + PD-M. Con: control; CP: cisplatin; PD: polydatin; LC3: autophagy microtubule-associated protein light chain 3; p62: sequestosome 1 (SQSTM1); BUN: blood urea nitrogen; Scr: serum creatinine; H&E: hematoxylin-eosin; TUNEL: terminal deoxynucleotidyl transferase dUTP nick-end labeling.

Based on the protective effect of PD on the autophagy flux of CP-treated cells in vitro, we further evaluated the effect of PD on autophagy flux in CP-AKI mice in vivo. To this end, different doses of PD (PD-L:20 mg/kg; PD-M: 40 mg/kg; PD-H: 80 mg/kg) were intraperitoneally injected 1 h before CP injection and then reinjected 24 h after CP injection. The CP-AKI mice were killed 48 h after CP injection (Figure 5(a)). Compared with the control mice, CP-AKI mice showed higher LC3 II and p62 expression in renal homogenates, suggesting that there was autophagy flux blocking in the kidneys of CP-AKI mice (Figures 5(b) and 5(c)). PD-M and PD-H significantly reversed the autophagy flux blocking in CP-AKI mice, but there was no apparent effect in PD-L (Figures 5(b) and 5(c)). Compared with the control mice, the body weight (Figure 5(d)) was decreased, and the kidney index (Figure 5(e)) was increased in CP-AKI mice, but they were remarkably reversed by PD treatment. Compared with CP alone, the administration of all three doses (PD-L, PD-M, and PD-H) reduced BUN (Figure 5(f)) and Scr (Figure 5(g)) in CP-AKI mice, in which PD-M was the best (reaching about 27.8% and 28.1% of the CP group). Likewise, histological examinations including H&E (Figures 5(h) and 5(i)) and TUNEL (Figures 5(j) and 5(k), Supplementary Table 1(a)) staining showed that all three doses of PD dramatically reduced tubular damage and cell apoptosis, and PD-M had the best effect. These results suggested that PD can accelerate the recovery of damaged autophagy flux and renal function in CP-AKI mice, and the dosage of 40 mg/kg PD is the best. Therefore, the subsequent experiments in this study were all performed following this treatment.

### 3.5. Chloroquine Eliminated the Nephroprotective Effect of PD on CP-AKI Mice in Terms of Recovery of Damaged Autophagy Flux, Anti-inflammation, and Antioxidative Stress

**Figure 6:**
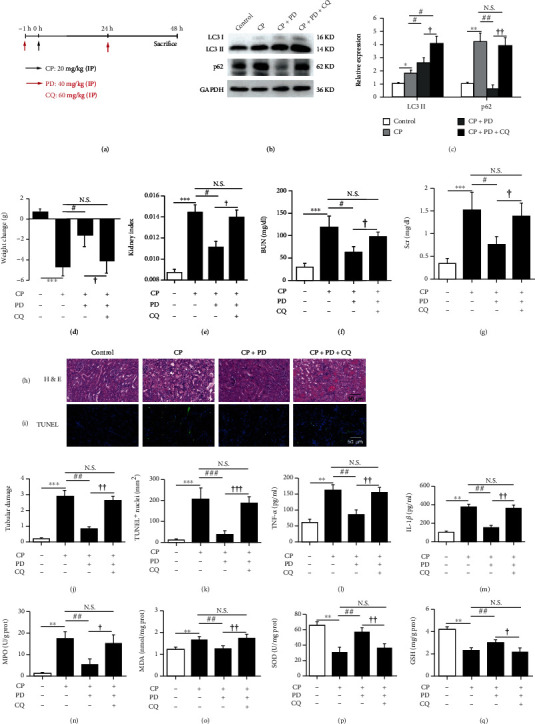
Inhibition of autophagy by CQ abolished the nephroprotective effect of PD on CP-AKI mice. (a) Mice pretreated with PD (40 mg/kg) or PD plus CQ (60 mg/kg) were administered with intraperitoneal injections of CP (20 mg/kg), and either PD or PD plus CQ was intraperitoneally reinjected at 24 h after CP injection. They were executed 48 h after the CP injection. (b and c) Western blot of LC3 and p62 in kidneys of CP-AKI mice that received PD (40 mg/kg), PD plus CQ (60 mg/kg), or vehicle (saline with 1% DMSO), and the semi-quantitative analysis of LC3 II/GAPDH and p62/GAPDH were shown. (d–g) Body weight changes, kidney index, BUN, and Scr were measured at 48 h after CP injection. (h and j) Histopathology analysis of the kidneys in CP-AKI mice was performed by H&E staining, and the tubular damage was graded. Scale bar = 50 *μ*m. (i and k) Representative TUNEL-stained sections of the kidney. Scale bar = 50 *μ*m. (l and m) Histologic levels of typical inflammatory cytokines including TNF-*α* and IL-1*β* in the kidneys were measured by ELISA. (n–q) Kidney tissue homogenates were evaluated by the assays of MPO (n), MDA (o), SOD (p), and GSH (q). ^∗^*P* < 0.05, ^∗∗^*P* < 0.01, and ^∗∗∗^*P* < 0.001 vs. control; ^#^*P* < 0.05, ^##^*P* < 0.01, and ^###^*P* < 0.001 vs. CP; ^†^*P* < 0.05, ^††^*P* < 0.01, and ^†††^*P* < 0.001 vs. CP + PD. CP: cisplatin; PD: polydatin; LC3: autophagy microtubule-associated protein light chain 3; p62: sequestosome 1 (SQSTM1); BUN: blood urea nitrogen; Scr: serum creatinine; HE: hematoxylin-eosin; TUNEL: terminal deoxynucleotidyl transferase dUTP nick-end labeling; ELISA: enzyme-linked immunosorbent assay; TNF-*α*: tumor necrosis factor-*α*; IL-1*β*: interleukin-1*β*; MPO: myeloperoxidase; MDA: malondialdehyde, SOD: superoxide dismutase; GSH: glutathione.

The inhibitory effect of CQ on renal autophagy flux in CP-AKI mice in vivo has been widely confirmed [3]. To figure out the role of autophagy flux in the nephroprotection of PD, PD (40 mg/kg) and CQ (60 mg/kg) were intraperitoneally injected into CP-AKI mice 1 h before CP administration and then reinjected 24 h after CP administration (Figure 6(a)). As shown in Figures 6(b) and 6(c), in CP-AKI mice, the PD-increased degradation of p62 (a selective substrate of autophagy) was inhibited by CQ, and the expression of LC3 II was increased, suggesting that blocking lysosomal degradation by CQ abolished the recovery effect of PD on autophagy flux. Concomitantly, the protective effect of PD on body weight (Figure 6(d)), kidney index (Figure 6(e)), BUN (Figure 6(f)), Scr (Figure 6(g)), renal tubular damage (Figures 6(h) and 6(j)), and cell apoptosis (Figures 6(i) and 6(k), Supplementary Table 1(b)) in CP-AKI mice was reversed by CQ.

In AKI, besides apoptosis, the mutual crosstalk of autophagy with inflammation and oxidative stress has also been confirmed [36]. CP-AKI mice had significantly increased TNF-*α* (Figures 6(l)) and IL-1*β* (Figure 6(m)) levels in the kidney, compared to the control group. PD reduced the levels of TNF-*α* and IL-1*β* in the kidneys of CP-AKI mice, which was abolished by combined CQ treatment (Figures 6(l) and 6(m)). To validate the potential effect of PD on antioxidative stress in CP-AKI, we detected the activity of MPO (Figure 6(n)) and SOD (Figure 6(p)) and the contents of MDA (Figure 6(o)) and GSH (Figure 6(q)) in the kidneys, respectively. Compared with the control mice, the MPO activity and MDA content were significantly increased in CP-AKI mice, which were reversed in PD group, but the beneficial effect of PD was abolished by CQ (Figures 6(n) and 6(o)). Likewise, CQ abolished the antioxidant effect of PD in CP-AKI mice, showing the decrease of SOD activity and GSH content (Figures 6(p) and 6(q)). These results suggested that the nephroprotective effect of PD on CP-AKI mice was at least partly related to the recovery of autophagy flux.

### 3.6. Inhibition of SIRT6 by OSS-128167 Eliminated the Nephroprotective Effect of PD on CP-AKI Mice in Terms of Recovery of Damaged Autophagy Flux, Anti-inflammation, and Antioxidative Stress

**Figure 7:**
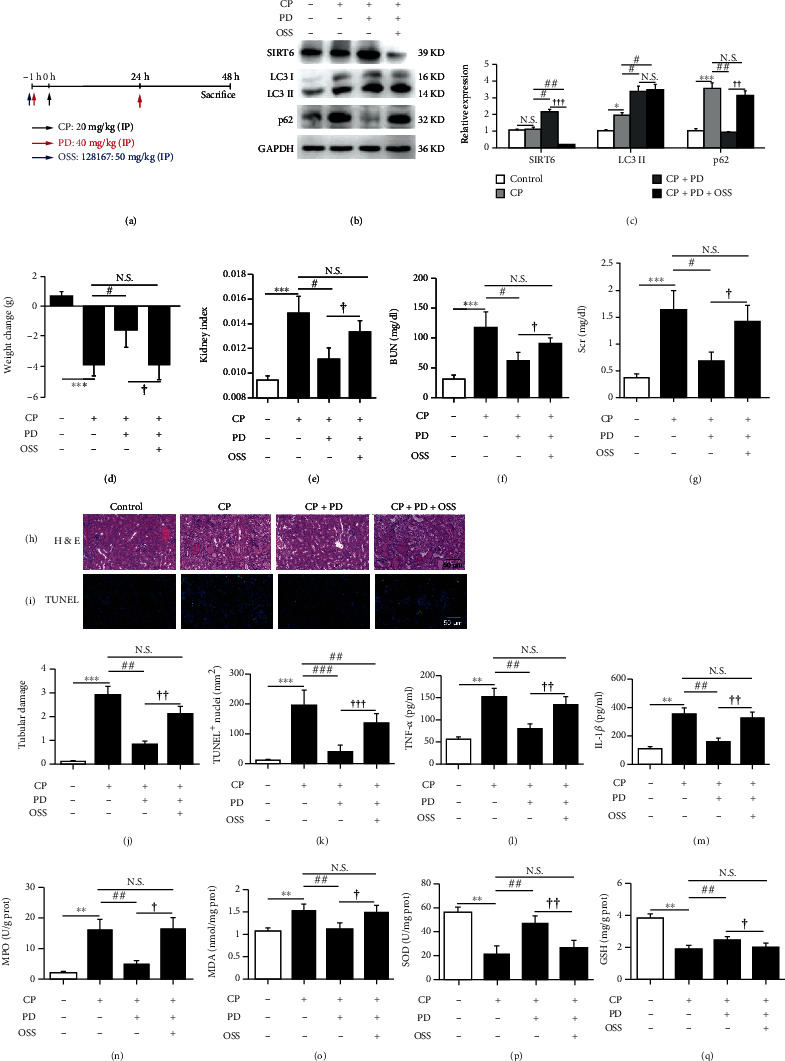
Inhibition of SIRT6 by OSS-128167 reversed the recovery of damaged autophagy flux and renal function of PD on CP-AKI mice. (a) Mice pretreated with PD (40 mg/kg) or PD plus OSS-128167 (50 mg/kg) were administered with intraperitoneal injections of CP (20 mg/kg), and PD was intraperitoneally reinjected at 24 h after CP injection. They were executed 48 h after the CP injection. (b and c) Western blot of SIRT6, LC3, and p62 in the kidneys of CP-AKI mice that received PD (40 mg/kg), PD plus OSS-128167 (50 mg/kg), or vehicle (saline with 1% DMSO), and the semiquantitative analysis of SIRT6/GAPDH, LC3 II/GAPDH, and p62/GAPDH were shown. (d–g) Body weight changes, kidney index, BUN, and Scr were measured at 48 h after CP injection. (h and j) Histopathology analysis of the kidneys in CP-AKI mice was performed by H&E staining, and the tubular damage was graded. Scale bar = 50 *μ*m. (i and k) Representative TUNEL-stained sections of the kidney. Scale bar =50 *μ*m. (l and m) Histologic levels of typical inflammatory cytokines including TNF-*α* and IL-1*β* in the kidneys were measured by ELISA. (n–q) Kidney tissue homogenates were evaluated by the assays of MPO (n), MDA (o), SOD (p), and GSH (q). ^∗^*P* < 0.05, ^∗∗^*P* < 0.01, and ^∗∗∗^*P* < 0.001 vs. control; ^#^*P* < 0.05, ^##^*P* < 0.01, and ^###^*P* < 0.001 vs. CP; ^†^*P* < 0.05, ^††^*P* < 0.01, and ^†††^*P* < 0.001 vs. CP + PD. CP: cisplatin; PD: polydatin; OSS: OSS-128167; SIRT6: sirtuin 6; LC3: autophagy microtubule-associated protein light chain 3; p62: sequestosome 1 (SQSTM1); BUN: blood urea nitrogen; Scr: serum creatinine; HE: hematoxylin-eosin; TUNEL: terminal deoxynucleotidyl transferase dUTP nick-end labeling; ELISA: enzyme-linked immunosorbent assay; TNF-*α*: tumor necrosis factor-*α*; IL-1*β*: interleukin-1*β*; MPO: myeloperoxidase; MDA: malondialdehyde, SOD: superoxide dismutase; GSH: glutathione.

To further evaluate the effect of SIRT6 on the nephroprotection of PD (40 mg/kg) in CP-AKI mice in vivo, the mice were orally administered OSS-128167 (50 mg/kg) through gavage 1 h before CP (20 mg/kg) injection (Figure 7(a)). WB results showed that PD significantly promoted SIRT6 expression in CP-AKI mice, and this effect was clearly inhibited by OSS-128167 (Figures 7(b) and 7(c)). Meanwhile, OSS-128167 also clearly abolished the recovery effect of PD on the autophagy flux in the kidneys of CP-AKI mice (Figures 7(b) and 7(c)). In addition, OSS-128167 also diminished the protective effect of PD on body weight (Figure 7(d)), kidney index (Figure 7(e)), BUN (Figure 7(f)), Scr (Figure 7(g)), renal tubular damage (Figures 7(h) and 7(j)), and cell apoptosis (Figures 7(i) and 7(k), Supplementary Table 1(c)) in CP-AKI mice. Furthermore, OSS-128167 also significantly inhibited the anti-inflammatory and antioxidative stress effects of PD in CP-AKI mice, which showed increase in the levels of TNF-*α* (Figure 7(l)) and IL-1*β* (Figure 7(m)), the MPO activity (Figure 7(n)) and MDA content (Figure 7(o)), and decrease in the SOD activity (Figure 7(p)) and GSH content (Figure 7(q)). These data suggested that the protective effect of PD on impaired autophagy flux and renal function in CP-AKI mice was at least partially related to the activation of SIRT6.

## 4. Discussion

On account of its nephrotoxicity, including AKI, CP is limited in the treatment of malignant tumors [1]. It has been proved that autophagy flux blocking is one of the mechanisms of CP nephrotoxicity, but so far there is no specific drug for AKI. We have recently confirmed the nephroprotective effect of PD in CP-AKI [4], but the role of PD in the damaged autophagy flux has not been reported in CP-AKI mice. In this study, the correlation between autophagy flux and SIRT6 in the nephroprotection of PD was investigated in in vivo and in vitro models of CP-AKI, with the following highlights: (1) It was first confirmed that PD could alleviate CP-AKI by restoring autophagy flux; (2) it was confirmed for the first time that the inhibition of SIRT6 pathway reverses the recovery effect of PD on CP-blocked autophagy flux, suggesting the potential effect of SIRT6-mediated autophagy flux on the nephroprotection of PD in CP-AKI.

Autophagy is a lysosomal degradation pathway, which acts a nephroprotective role under normal physiological conditions and when the kidney is exposed to injuries or toxins, such as CP [2]. The pharmacologic and genetic inhibition and activation of autophagy can increase and reduce renal tubular injury during CP treatment, respectively [2]. This study showed that the autophagy flux was blocked in CP-treated HK-2 cells and CP-AKI mice, which was characterized by the increase of LC3 II and p62 in WB, and the decrease of autophagolysosome/autophagosome ratio in mRFP-GFP confocal test, which were consistent with other studies [37, 38]. The autophagy flux blocking and cell apoptosis induced by CP in a dose-dependent and time-dependent manner were further aggravated by CQ and alleviated by RAP, suggesting that autophagy flux blocking may be associated with CP nephrotoxicity.

Polydatin, a natural polyphenol plant extract, has been proved to have a nephroprotective effects in different AKI and chronic kidney disease (CKD) models [21]. Deng et al. proved an important role of PD in autophagy by activating SIRT1-mediated Beclin1 deacetylation in sepsis-induced AKI [5]. Gu et al. showed that PD ameliorated autophagy imbalance in an mTORC1-dependent manner during fructose-induced podocyte injury [25]. The renal protective effect of PD in CP-AKI has recently been confirmed by our research team [4], but it is not clear whether PD can resist CP nephrotoxicity by regulating autophagy. The results of this study clearly showed that PD can restore CP-induced autophagy flux blocking in both CP-treated HK-2 cells in vitro and CP-AKI animals in vivo, which were manifested by the increase in LC3 II and autophagolysosome/autophagosome ratio, and the decrease in p62 accumulation; however, this effect was blocked by CQ. Concomitantly, renal protective effects of PD on CP-AKI, including antiapoptosis, anti-inflammation, and antioxidative stress, were also revered by CQ, indicating that PD could at least partially protect mice against CP-AKI by restoring autophagy flux.

Cisplatin can cause multiple forms of cellular stress, such as oxidative stress, endoplasmic reticulum stress, mitochondrial damage, and mitophagy, which may be related to autophagy activation in CP-AKI [2]. It has been widely reported the role of energy signaling pathways mediated by mTOR, AMPK, and NAD^+^ metabolism in the regulation of autophagy in CP nephrotoxicity [1]. Sirtuin 6 (SIRT6) belongs to NAD^+^-dependent deacetylase, which is widely expressed in mammalian organs, and regulates multiple biological processes, including inflammatory response, oxidative stress, telomere homeostasis, and autophagy [31]. The protective effect of SIRT6 activating autophagy has been proved in different organ injuries, including I/R injury [39–41], diabetes [42–45], and sepsis [19, 46, 47]. Several studies have recently demonstrated the renal protective effect of SIRT6-induced autophagy in sepsis-induced AKI [47], hypertensive cardiorenal injury [48], podocyte injury [45], and cadmium-induced renal damage [49], respectively. Li et al. [32] confirmed that overexpression of SIRT6 attenuated CP-AKI by inhibiting extracellular signal-regulated kinase 1/2 signaling; Fan et al. [50, 51] confirmed that isoorientin and daphnetin can alleviate CP-AKI through antioxidative stress and antiapoptosis via activating the SIRT1/SIRT6/nuclear factor erythroid 2-related factor 2 pathway, but the relationship between SIRT6 and autophagy has not been reported in CP-AKI. A recent report shows that PD protects against septic myocardial injury by activating SIRT6-mediated autophagy [19]. Therefore, based on the recovery effect of PD on autophagy flux blocking in CP-AKI confirmed in this study, we further evaluated whether SIRT6 was involved. As expected, in the CP-AKI models in vivo and in vitro, OSS-128167, an inhibitor of SIRT6, not only inhibited SIRT6 expression promoted by PD, but also blocked the role of PD in the recovery of autophagy flux. Meanwhile, the inhibition of SIRT6 also weakened the renal protective effects of PD on CP-AKI, including antiapoptosis, anti-inflammation, and antioxidative stress, which suggested that PD was at least partially protected from CP-AKI through the SIRT6-mediated activation of autophagy.

Autophagy is protective during CP-AKI, but it is considered as a double-edged sword in cancer therapy [1, 3]. The strategy of targeting autophagy in CP chemotherapy must consider the effects on both healthy and tumorous tissues, including the kidneys. Besides the protective effect on different organ injuries, the antitumor role of PD through different pathological effects including autophagy has also been confirmed [5, 6]. Clinically, PD has been used to treat patients with chronic alcoholism [16], irritable bowel syndrome [17], and interstitial cystitis/bladder pain syndrome [18]. Surprisingly, PD has recently been proposed as a potential natural active drug for the treatment of coronavirus disease 2019 [52–57]. All these evidences suggest that PD may be a promising option for patients with AKI during CP chemotherapy. It is imperative to further explore the mechanism of PD in AKI and CKD, including autophagy.

## 5. Conclusions

**Figure 8:**
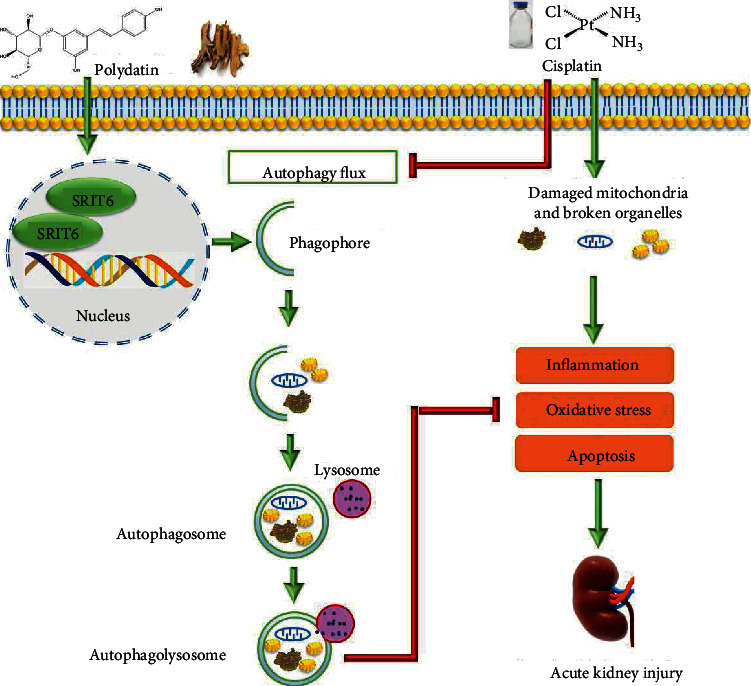
Cisplatin causes damage of mitochondria and other organelles, leading to oxidative stress, inflammatory response, and cell apoptosis. PD regulates autophagy level by upregulating SIRT6 expression. This increase in autophagy flux phagocytizes the damaged or dysfunctional organelles and inhibits oxidative stress, inflammatory cascade, and cell apoptosis, thus alleviating cisplatin-induced AKI.

Collectively, our study demonstrated for the first time that PD boosts autophagy flux through SIRT6 upregulation and protects renal tubular epithelial cells from oxidative stress, inflammatory response, and apoptosis, thus alleviating cisplatin-induced AKI (Figure 8). PD seems a promising inducer for restoration of autophagy flux for AKI. Furthermore, PD could be considered as a renoprotective natural compound in cisplatin-induced AKI, although further studies are required to confirm its beneficial effects in CKD development after CP chemotherapy.

## Data Availability

All data related to this paper may also be requested from the corresponding authors (email: xjsnlhb@fmmu.edu.cn).

## Abbreviations

AKI: Acute kidney injury
BUN: Blood urea nitrogen
Cis-AKI: Cisplatin-induced acute kidney injury
CKD: Chronic kidney disease
CLSM: Confocal laser scanning microscope
CQ: Chloroquine
ELISA: Enzyme-linked immunosorbent assay
FACS: Fluorescence-activated cell sorting
GSH: Glutathione
IL-1*β*: Interleukin-1*β*
I/R: Ischemia/reperfusion
I/R-AKI: Ischemia/reperfusion-induced acute kidney injury
LC-3: Autophagy microtubule-associated protein light chain 3
MDA: Malondialdehyde
MPO: Myeloperoxidase
NAD^+^: Nicotinamide adenine dinucleotide
PD: Polydatin
PD-H: High-dose polydatin
PD-L: Low-dose polydatin
PD-M: Moderate-dose polydatin
PI: Propidium iodide
p62: Sequestosome 1 (SQSTM1)
RAP: Rapamycin
Scr: Serum creatinine
SIRT6: Sirtuin 6
SOD: Superoxide dismutase
TNF-*α*: Tumor necrosis factor-*α*
TUNEL: Terminal deoxynucleotidyl transferase dUTP nick-end labeling
WB: Western blot.

## Supplementary Materials

Supplementary Materials Supplementary Table 1: number of TUNEL-positive cells in the kidneys of mice in various treatment groups.
