# Supplementary material for: Polydatin Attenuates Cisplatin-Induced Acute Kidney Injury via SIRT6-Mediated Autophagy Activation
Source: Oxid Med Cell Longev. 2022 Sep 16;2022:9035547. doi: 10.1155/2022/9035547 (PMC9507782; doi:10.1155/2022/9035547)
Supplement: Supplementary Materials — Supplementary Table 1: number of TUNEL-positive cells in the kidneys of mice in various treatment groups. [file 9035547.f1.docx]

**Supplementary** **Table 1: Number of TUNEL positive cells in kidneys of mice in various treatment groups.**

| Groups | n | Number of TUNEL positive cells (mm^2^) | |
| --- | --- | --- | --- |
| Group (a) |  |  |  |
| Control | 6 | 11.33±3.266 |  |
| CP | 8 | 201.25±46.32 | *** |
| CP+PD-L | 8 | 114.50±35.82 | *** ^## †††^ |
| CP+PD-M | 8 | 40.38±15.45 | **^###^ |
| CP+PD-H | 8 | 71.25±16.92 | *** ^### ††^ |
| Group (b) |  |  |  |
| Control | 6 | 11.00±3.098 |  |
| CP | 8 | 212.13±45.37 | *** |
| CP+PD-M | 8 | 43.25±16.67 | ** ^###^ |
| CP+PD-M+CQ | 8 | 189.50±29.43 | *** ^†††^ |
| Group (c) |  |  |  |
| Control | 6 | 12.00±4.099 |  |
| CP | 8 | 198.25±45.24 | *** |
| CP+PD-M | 8 | 42.13±16.13 | ** ^###^ |
| CP+PD-M+OSS-128167 | 8 | 136.75±28.00 | *** ^## †††^ |

CP: cisplatin; PD: polydatin; PD-L: CP + PD low dose; PD-M: CP + PD middle dose; PD-H: CP + PD high dose; CQ: chloroquine; TUNEL: terminal deoxynucleotidyl transferase dUTP nick-end labeling. **P* < 0.05, ***P* < 0.01, ****P* < 0.001 vs. control; ^#^*P* < 0.05, ^##^*P* < 0.01, ^###^*P* < 0.001 vs. CP; **^†^***P* < 0.05, **^††^***P* < 0.01, **^†††^***P* < 0.001 vs. CP+PD-M.
